# A phase I and pharmacokinetic study of MAG-CPT, a water-soluble polymer conjugate of camptothecin

**DOI:** 10.1038/sj.bjc.6600516

**Published:** 2002-09-09

**Authors:** N E Schoemaker, C van Kesteren, H Rosing, S Jansen, M Swart, J Lieverst, D Fraier, M Breda, C Pellizzoni, R Spinelli, M Grazia Porro, J H Beijnen, J H M Schellens, WW ten Bokkel Huinink

**Affiliations:** Department of Medical Oncology, Antoni van Leeuwenhoek Hospital/The Netherlands Cancer Institute, Plesmanlaan 121, 1066 CX, Amsterdam, The Netherlands; Department of Pharmacy and Pharmacology, The Netherlands Cancer Institute/Slotervaart Hospital, Louwesweg 6, 1066 EC, Amsterdam, The Netherlands; Global Drug Metabolism, Pharmacia, Viale Pasteur 10, 20014 Nerviano, Milan, Italy; Clinical Research Oncology, Pharmacia, Viale Pasteur 10, 20014 Nerviano, Milan, Italy; Division Drug Toxicology, Faculty of Pharmacy, Utrecht University, Sorbonnelaan 16, 3584 CA, Utrecht, The Netherlands

**Keywords:** polymer, camptothecin, pharmacokinetics, MAG-CPT, controlled release, drug conjugate

## Abstract

Polymeric drug conjugates are a new and experimental class of drug delivery systems with pharmacokinetic promises. The antineoplastic drug camptothecin was linked to a water-soluble polymeric backbone (MAG-CPT) and administrated as a 30 min infusion over 3 consecutive days every 4 weeks to patients with malignant solid tumours. The objectives of our study were to determine the maximal tolerated dose, the dose-limiting toxicities, and the plasma and urine pharmacokinetics of MAG-CPT, and to document anti-tumour activity. The starting dose was 17 mg m^−2^ day^−1^. Sixteen patients received 39 courses at seven dose levels. Maximal tolerated dose was at 68 mg m^−2^ day^−1^ and dose-limiting toxicities consisted of cumulative bladder toxicity. MAG-CPT and free camptothecin were accumulated during days 1–3 and considerable amounts of MAG-CPT could still be retrieved in plasma and urine after 4–5 weeks. The half-lives of bound and free camptothecin were equal indicating that the kinetics of free camptothecin were release rate dependent. In summary, the pharmacokinetics of camptothecin were dramatically changed, showing controlled prolonged exposure of camptothecin. Haematological toxicity was relatively mild, but serious bladder toxicity was encountered which is typical for camptothecin and was found dose limiting.

*British Journal of Cancer* (2002) **21**, 608–614. doi:10.1038/sj.bjc.6600516 www.bjcancer.com

© 2002 Cancer Research UK

## 

Camptothecin and analogues are an important class of potent antineoplastic drugs (Kehrer *et al*, 2001). Camptothecin showed strong cytotoxicity against a variety of tumour types *in vitro* and *in vivo* (Wall and Wani, 1996; Chabot, 1997). However the water soluble salt of camptothecin (carboxylate form) induced severe toxicity and low tumour response rates in early phase I and II studies. Dose limiting toxicity consisted of severe cumulative haematological toxicity at cumulative doses of >100 mg m^−2^ camptothecin. Major other toxicities were vomiting, diarrhoea, and chemical or haemorrhagical cystitis which were often formidable and unpredictable (Moertel *et al*, 1972; Muggia *et al*, 1972). In the 1990s it was discovered that the topoisomerase I enzyme (TopI) was the cellular target of camptothecin and that the lactone function is essential for anti-tumour activity (Slichenmyer *et al*, 1993; Rivory and Robert, 1995; Wall and Wani, 1996). However, further clinical development of camptothecin was hampered by its unfavourable physical properties. Camptothecin is extremely insoluble in water and in plasma the pH favours the conversion of the lactone into the inactive carboxylate form (Slichenmyer *et al*, 1993). The half-life of the lactone form in plasma is 12 min and within 2 h 99% has been hydrolysed into its carboxylate form (Slichenmyer *et al*, 1993; Rivory and Robert, 1995; Chabot, 1997). A new approach to overcome these drawbacks is the water soluble drug-polymer construct MAG-CPT. MAG-CPT consists of a copolymer backbone (methacryloylglycinamide, MAG) to which camptothecin is covalently conjugated at the C-20 position via an amino acid spacer (Figure 1

**Figure 1:**
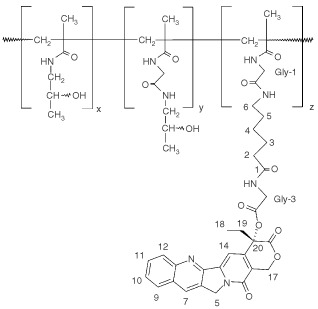
Chemical structure of MAG-CPT.

). The release of free camptothecin into the blood stream is now dependent on the rate of pH mediated (and enzyme mediated) esterolytic cleavage of MAG-CPT (Conover *et al*, 1999). An important potential advantage of polymeric drugs is the so called ‘enhanced permeability and retention’ (EPR) effect (Seymour, 1992). Whereas systemic exposure of the cytotoxic drug is diminished by attachment to the polymeric carrier, an accumulation takes place at the tumour site due to the increased vasopermeability of many tumours. In pre-clinical experiments decreased toxicity of normal tissue and improved anti-tumour efficacy probably due to prolonged intra-tumour retention could be demonstrated (Caiolfa *et al*, 2000; Conover *et al*, 1998). To study the pharmacokinetics of MAG-CPT polymer bound drug as well as free drug should be considered. Objectives of our study were (1) to determine the maximal tolerated dose (MTD) and dose-limiting toxicities (DLT) of MAG-CPT, (2) to determine the plasma and urine pharmacokinetic profile of MAG-CPT and free camptothecin and (3) to document any anti-tumour activity.

## PATIENTS AND METHODS

### Eligibility criteria

Patients with histological or cytological proof of malignant solid tumour, for whom no recognised therapy was available for their disease, were eligible. Other eligibility criteria included age ⩾18 years, World Health Organisation (WHO) performance status ⩽2, and estimated life expectancy ⩾12 weeks (Who, 1979). Previous chemotherapy had to be discontinued for at least 4 weeks before entry into the study, or 6 weeks in case of mitomycin C or nitrosourea. Patients had to be recovered from all acute toxic effects (excluding alopecia and grade 1 neurotoxicity) from any prior therapy. Patients had to have acceptable bone marrow function defined as absolute neutrophil count (ANC) ⩾2000 μl^−1^ and platelet count ⩾100 000 μl^−1^; adequate hepatic function defined as serum bilirubin, alanine-aminotransferase (ALAT) and aspartate-aminotransferase (ASAT) within normal limits or when patients had liver metastases ALAT and ALAT ⩽five times under normal limits (National Cancer Institute Common Toxicity Criteria (NCI-CTC) grade 2), and bilirubin ⩽NCI-CTC grade 1; and adequate renal function defined as serum creatinine ⩽1.5 mg dl^−1^ or 133 μmol l^−1^ and calculated creatinine clearance according to described formulas (Lott and Hayton, 1978; Traub and Johnson, 1980; Nci, 1998). Ineligibility criteria included a history of treatment with other camptothecins and/or more than two prior chemotherapies for metastatic disease, intensive ablative regimens requiring peripheral stem cell transplantation or autologous bone marrow transplantation, prior radiotherapy to more than 25% of the bone marrow bearing areas, known brain or leptomeningeal disease, severe systemic disease, haematological malignancies, known hepatitis B or HIV positive or AIDS-related illness. The study protocol was approved by the Medical Ethics Committee of the hospital, and all patients gave written informed consent.

### Toxicity and response evaluation

Pre-treatment evaluation included a complete medical history and complete physical examination. Before each course, a complete physical examination was performed. Furthermore, blood chemistry and haematology profiles were checked, and urinalysis was done. Before, immediately after and 1 h after each MAG-CPT infusion the blood pressure was measured. Complete blood cell counts were repeated weekly until occurrence of first grade 1 leukocytopenia or neutropenia and twice a week thereafter, and blood chemistries and urinalysis were done weekly. In addition, urea, creatinine and electrolytes were measured 24 h after the first dose of MAG-CPT in each cycle. Electrocardiograms and tumour measurements (by physical examination, chest X-ray, other radiological investigations or ultrasound) were performed every three cycles. All toxicities were graded according to the NCI-CTC (Nci, 1998). Dose-limiting toxicities were defined as any of the following events occurring during the first treatment cycle and attributable to MAG-CPT: (1) grade 4 neutropenia lasting 5 days or of any duration associated with infection of severity ⩾3, (2) febrile neutropenia, (3) grade 3 or 4 thrombocytopenia or (4) grade 3 or 4 non-haematological toxicity (grade 2 for neurotoxicity) excluding nausea/vomiting responsive to treatment, fatigue and alopecia. The maximum tolerated dose (MTD) was defined as the dose at which two of three or two of six patients experienced DLT. The next lower dose-level below the MTD was the recommended dose for phase II studies. Responses were determined according to the WHO criteria (Who, 1979).

### Dose escalation

Dose escalation was performed according to a modified Fibonacci series in which dose increments for succeeding levels are defined as 100, 67, 50 and 40% followed by 33% for all subsequent levels. According to Simon *et al* (1997) an accelerated titration design was used by entering one patient per dose-level. Before entering a new patient at the next dose-level, the previous patient had to be followed for 4 weeks. Treatment cycles were repeated every 28 days provided patients had sufficiently recovered from any drug-related toxicity associated with the previous course (non-haematological toxicity grade 1, alopecia excluded, and return of blood counts to 2000 μl^−1^ neutrophils and 100 000 μl^−1^ platelets).

### Drug administration

MAG-CPT was supplied by Pharmacia & Upjohn (Milan, Italy) in vials containing 0.5 g of active ingredient (50 mg of camptothecin equivalent) and lactose, polysorbate 80 as excipients and 1 N sodium hydroxide as pH adjusting agent. All doses are expressed as camptothecin equivalent. The content of each vial was reconstituted with 5 or 10 ml of 0.9% sodium chloride and subsequently diluted. MAG-CPT was administered intravenously through a central or peripheral venous access device over 30 min by a syringe pump. Before and after infusion the venous access was flushed with a small volume of 0.9% sodium chloride.

### Pharmacokinetics

Complete pharmacokinetic studies were performed during the first treatment course. On day 1 blood samples (5 ml each), drawn from an indwelling intravenous cannula placed in the arm contralateral to the arm receiving MAG-CPT, were collected in cooled heparinised tubes pre-infusion, and at 15, 30, 40, 60, and 90 min, and 2.5 and 5.5 h after the start of the infusion. On day 2, samples were collected prior to infusion and at 30 min, and 4.5 and 8.5 h after the start of the infusion. On day 3, samples were collected prior to infusion and at 30 and 50 min, and 3.5, 6.5, 24 and 48 h after the start of the infusion. Additional samples were collected weekly thereafter until start of next cycle. Blood samples were immediately immersed in ice-water at the bed-side. Plasma was obtained by refrigerated centrifugation of the samples (5 min; 3000 **g**, 4°C). For the determination of free camptothecin, 0.25 ml of plasma was added to 0.75 ml 8.5% phosphoric acid and subsequently mixed on a whirl mixer for 10 s. Plasma and plasma phosphoric acid mixture were stored in polypropylene tubes and immediately put in a dry ice/ethanol bath and thereafter stored by −70°C until analysis. Urine was collected at days 1, 2 and 3 with timed collections (0–8, 8–24 h) and 0–24 h at day 4 of the first course. A sample of each urine portion was taken and stored in a propylene tube at −70°C until analysis. Additional urine-samples were collected weekly thereafter until start of the next cycle.

Plasma levels of MAG-CPT and free camptothecin were determined using a validated reversed-phase HPLC system with fluorescence detection by Pharmacia and Upjohn (Milan, Italy) (Schoemaker *et al*, 2001). Total camptothecin levels were determined after hydrolysis of MAG-CPT and free camptothecin was extracted from acidified plasma before determination. Concentrations of polymer-bound camptothecin were calculated by subtraction of free from total camptothecin. The lower limit of quantification (LLOQ) of the methods were 100 ng ml^−1^ for total and 1.1 ng ml^−1^ for free CPT using 50 and 250 μl plasma, respectively. Determination of levels of total MAG-CPT in urine was performed with a similar method. The LLOQ in urine for total camptothecin was 100 ng ml^−1^. The pharmacokinetic parameters were calculated using a model-independent method using WinNonlin package (version 2.1, Scientific Consulting Inc., 1996). Carrier-bound CPT levels were calculated by subtracting free CPT to total CPT. For the pharmacokinetic calculations the actual sampling times were used. Pre-dose plasma levels were put equal to zero. After MAG-CPT administration, the maximum plasma concentration (C_max_) and the corresponding time were taken directly from the raw data for each subject. The terminal rate constant *k* was determined by log-linear regression analysis of the terminal phase of the plasma concentration-time curve. The choice of number of points of the terminal phase was based on visual inspection of the data. The area under the plasma concentration-time curve between 0 and 96 h, AUC_0–96_, was estimated by the linear-logarithmic trapezoidal method from data points ranging from pre-dose until 96 h after dosing. The total AUC, AUC_∞_, was determined by the linear trapezoidal rule up to the last detectable concentration, beyond that time, extrapolation was performed from the last measured data point to infinity using *k* (C_last_ /*k*). Total body clearance of MAG-CPT from plasma (Cl) after intravenous administration was calculated as the total administered dose divided by AUC_∞_. The free to bound ratio of MAG-CPT (ratio_f/b_)was calculated by dividing the total AUC of free camptothecin by the total AUC of bound camptothecin times 100%. The terminal half-life (t_½_) was calculated as 0.693/*k*. The percentage of the administered dose recovered in urine over 4 days was calculated as the amount excreted in urine divided by the total administered dose. Total renal clearance (Cl_R_) of MAG-CPT was calculated as the amount excreted in urine unchanged divided by the AUC_0–96_ of bound CPT. Data are represented as mean±s.d.

### Statistical analysis

The Pearson correlation coefficient (*r*) was calculated between dose, the AUC of MAG-CPT and free camptothecin. Patient's, haematological, and biochemical parameters (including markers for hepatic and renal function) were correlated to pharmacokinetic parameters of MAG-CPT and free camptothecin using the non-parametric Spearman rank correlation test (r_s_) to investigate determinants in inter-patient pharmacokinetic variability. Statistical analysis was performed with SPSS (Statistical Package for Social Sciences, version 6.1 for Windows). The level of significance (*P*) was set at 0.05. All tests for significance were two-tailed.

### Pharmacokinetic-pharmacodynamic analysis

Relationships between pharmacokinetic parameters of bound and free camptothecin and categorical toxicity data (nausea, vomiting, and dysuria) were explored using the Spearman rank correlation test. Relationships between the dose and the AUC of MAG-CPT and free camptothecin and myelosuppression were explored using scatter plots of the AUC *vs* the percentage decrease in white blood cell (WBC) count, ANC, and platelet count of the first cycle. The percentage decrease in blood cells is defined as: (100 × (baseline count–nadir count))/baseline count. The data were fitted using (log)-linear and sigmoidal maximum effect (E_max_) models using the software package WinNonlin™ (version 3.0, Pharsight corporation). Only data obtained in the first cycle were used.

## RESULTS

### Patients and treatment

Sixteen patients received a total of 39 courses at seven dose-levels with a median of 2 (range 1–5) courses per patient. Patient characteristics are outlined in Table 1

**Table 1:**
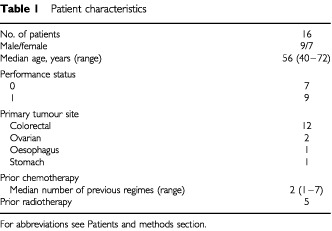
Patient characteristics

. The following dose-levels were evaluated: 17, 34, 57, 68, 85, 100, 130 mg m^−2^ day^−1^ for 3 days. Fifteen patients were assessable for toxicity during the first course. Fully evaluable pharmacokinetic data were available of 15 patients. One patient was not evaluable for toxicity and pharmacokinetics during the first course because of infusion leakage on the first day of treatment. No MAG-CPT or free camptothecin could be detected in his blood samples collected on this day. Four patients did not receive a second course according to this protocol, because of rapid progressive disease.

### Haematological toxicity

Haematological toxicity was rare with MAG-CPT administered in this schedule. Serious non dose limiting haematological toxicity was only encountered in one patient at dose-level 5 with one episode of grade 3 leukocytopenia combined with grade 4 neutropenia and in one patient at dose-level 7 with one episode of grade 3 neutropenia.

### Non-haematological toxicity

Bladder toxicity, consisting of sustained frequent dysuria and microscopic or macroscopic haematuria was dose limiting. In Table 2

**Table 2:**
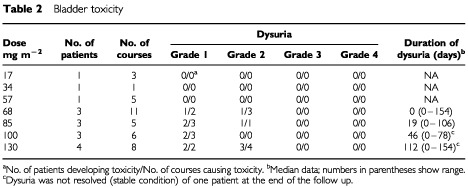
Bladder toxicity

the incidence and duration of dysuria is given because no adequate official grading system for this kind of toxicity is available. Dysuria and haematuria was only noted in patients treated at dose-level 4 (68 mg m^−2^ day^−1^) and higher. Symptoms generally started during the first or the second course and seemed initially mild to moderate. However some of the patients who received more than one course gradually developed very significant toxicity. One patient at dose-level 6 and two patients at dose-level 7 were treated after the onset of the symptoms with sodiumbicarbonate with minor results. Most patients had their symptoms gradually resolved after withdrawal of MAG-CPT. After evaluation of this apparently cumulative toxicity it was decided that it concerned a grade 3/4-type of toxicity and no more patients were to be treated with MAG-CPT at this schedule at these high dose-levels. Proposed dosing scheme for further investigation is 68 mg m^−2^ day^−1^ for 3 days every 4 weeks with coadministration of 4× 1000 mg day^−1^ carbohydrate starting 24 h before MAG-CPT because at a dose of 85 mg m^−2^ day^−1^ for 3 days every 4 weeks was the first dose level in which two of three patients experienced this kind of toxicity. Other non-haematological toxicity was generally mild (CTC ⩽2) except for one patient who experienced grade 3 nausea during one course. Main toxicity included nausea (10 patients), vomiting (five patients), diarrhoea (four patients), and fatigue (three patients).

### Efficacy

A number of 11 out of 16 patients were evaluable for response after two courses. The main reason for being not evaluable at first formal evaluation was early disease progression. No responses were found, however one patient with colon cancer had a stable disease which lasted for 62 days.

### Pharmacokinetics

A summary of the overall pharmacokinetics of MAG-CPT is listed in Tables 3

**Table 3:**
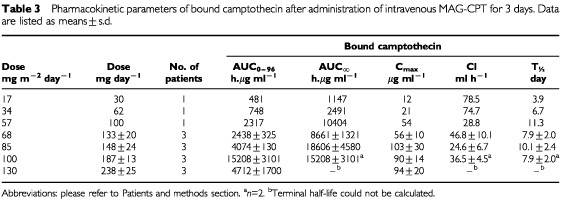
Pharmacokinetic parameters of bound camptothecin after administration of intravenous MAG-CPT for 3 days. Data are listed as means±s.d.

and 4

**Table 4:**
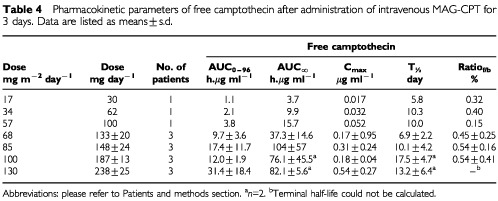
Pharmacokinetic parameters of free camptothecin after administration of intravenous MAG-CPT for 3 days. Data are listed as means±s.d.

**Figure 2:**
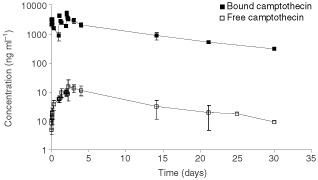
Mean plasma concentration-time curves of bound campto thecin and free camptothecin of patients treated with 68 mg m^−2^ day^−1^ MAG-CPT.

. Mean plasma concentration-time curves of bound and free camptothecin of patients treated with 68 mg m^−2^ day^−1^ MAG-CPT are presented in Figure 2

**Figure 3:**
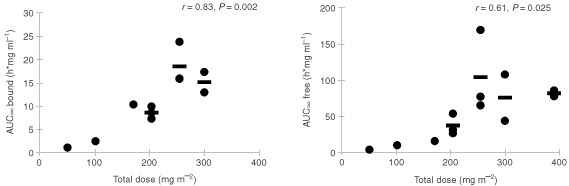
Plot of AUC_∞_ of bound camptothecin (left panel) and free camptothecin (right panel) as a function of the accumulative administered dose of MAG-CPT during course 1. Bars indicate mean values.

. Plasma levels of free camptothecin were approximately 100-fold lower than bound camptothecin. The mean terminal elimination half-life (t_½_) of bound camptothecin was 8.3±0.8 days (mean±s.d.) and of free camptothecin 10.2±4.8 days (mean±s.d.). There is no significant difference between the half-lifes of bound and free camptothecin (*P*=0.3). In Figure 3

the AUC of free and bound camptothecin is given as a function of the total dose (mg m^−2^) of MAG-CPT. Both the AUC of bound camptothecin (*r*=0.83, *P*=0.002) and free camptothecin (*r*=0.61, *P*=0.025) increase linearly with the MAG-CPT dose. The urine excretion data are summarised in Table 5

**Table 5:**
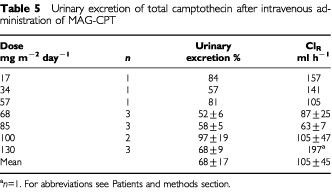
Urinary excretion of total camptothecin after intravenous administration of MAG-CPT

. The bulk of the dose excreted in the urine was recovered within the 3 days of MAG-CPT administration. At day 4 only small amounts of camptothecin were excreted in the urine (1–4% of total dose). At 4 weeks after administration of MAG-CPT concentrations of total camptothecin in urine of patients treated at dose levels 2–7 could still be measured (results not shown).

### Statistical analysis

Two renal function parameters, potassium and urea, were considered significant determinants of inter-patient pharmacokinetic variability (Table 6

**Table 6:**
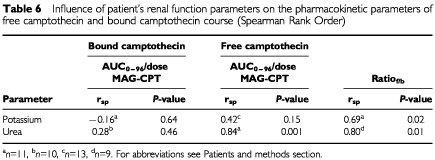
Influence of patient's renal function parameters on the pharmacokinetic parameters of free camptothecin and bound camptothecin course (Spearman Rank Order)

) after univariate analysis. Potassium levels were significant determinants of the ratio_f/b_. Urea levels were significant determinants of the ratio_f/b_ and AUC_∞_ of free camptothecin/dose MAG-CPT but not for the AUC_∞_ of bound camptothecin/dose MAG-CPT.

### Pharmacokinetic-pharmacodynamic analysis

**Figure 4:**
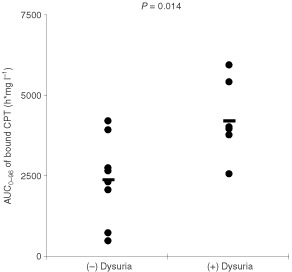
Plot of the AUC_0–96_ of bound camptothecin against the occurrence of dysuria during all courses of individual patients, *P* of difference is 0.014. Bars indicate mean values.

**Figure 5:**
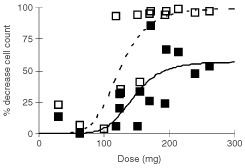
The percentage decreases in WBC (solid line) and ANC (dashed line) during the first course *vs* the daily dose. The lines indicate the best fit of the data to the sigmoidal maximal effect pharmacodynamic model (coefficient of correlation is 0.75 and 0.84 for WBC and ANC, respectively).

Patients with dysuria in one or more courses had significant higher AUC_0–96_ of bound camptothecin compared to patients without dysuria (*P*=0.014, Figure 4

). The AUC_∞_ of free camptothecin and the dose of MAG-CPT was significantly correlated with the decrease in ANC, platelets, and WBC. Furthermore, a significant correlation was found between the AUC_∞_ of bound camptothecin with the decrease in ANC. The decrease in WBC and ANC was most significant correlated with the dose (D) of MAG-CPT and this relation was best fit to a sigmoidal E_max_ model (parameters: E_max_=53.3%; D_50_=140 mg; γ=7.4, and E_max_=100%; D_50_=123 mg; γ=5.6 for WBC and ANC, respectively) (Figure 5

). The decrease in platelets was most significant linearly correlated with the dose of MAG-CPT (*r*=0.63, *P*<0.011), however the data did not fit to a sigmoidal E_max_ model (results not shown).

## DISCUSSION AND CONCLUSION

Over the past few decades significant advances have been made in technologies aimed at enhancing the pharmacological distribution properties of anti-cancer drugs. Synthetic drug-polymers are developed to control release and targeting of cytostatics. Recently the first clinical experiences were described of two copolymer constructs, with conjugated doxorubicin (PK-1) and paclitaxel (PNU 166945) (Vasey *et al*, 1999; Meerum Terwogt *et al*, 2001). PK-1 showed anti-cancer activity and the polymer-drug conjugation was able to decrease doxorubicin dose-limiting toxicities while no polymer associated toxicity was observed. The compound is currently tested in a phase II clinical trial. The phase I study with polymer bound paclitaxel was aborted prematurely due to accumulative and probable irreversible neurotoxicity found in a 13-week rodent study conducted at the same time. However, toxicity of polymer bound paclitaxel in patients at the achieved dose-levels was generally mild while activity was observed in one patient with breast cancer. We performed a clinical phase I study with MAG-CPT, a HPMA polymer conjugate of CPT (Figure 1). The starting dose of 17 mg m^−2^ day^−1^ for 3 days (i.e. 51 mg m^−2^) every 4 weeks was based on animal data. The dose corresponds with 1 out of 3 of the NOEL (no observed effective level, 1.5 mg kg day^−1^) in the dog treated i.v. with this dose for 5 consecutive days (total dose is 7.5 mg kg^−1^ or 145 mg m^−2^). Studies in animals demonstrated that MAG-CPT is better tolerated when given on a split-dose basis compared to a single administration. Three consecutive days of intravenous treatment has been selected as a safe and convenient therapy modality, which has the advantage to minimise patient's hospitalisation. The highest administered dose of MAG-CPT to patients in this phase I study was 130 mg m^−2^ day^−1^ for 3 days every 4 weeks. We found, however, no activity of MAG-CPT but the number of patients evaluable for efficacy in this study was very low and the patients participating in this study were heavily pre-treated.

Haematological toxicity of MAG-CPT at the investigated doses and schedule was mild. In addition, haematological toxicity was related to the administered dose. Unfortunately, bladder problems including dysuria and microscopic and sometimes macroscopic haematuria proved to be dose-limiting. These toxicities were only observed at the dose-levels of 68 mg m^−2^ day^−1^ and higher. In pre-clinical studies with MAG-CPT in rodents and dogs no bladder toxicity was observed (Wall and Wani, 1996; Chabot, 1997). In previous clinical studies with the sodium salt of camptothecin dose limiting toxicity consisted of haematological depression. But also haemorrhagical and chemical cystitis of the bladder was observed (Moertel *et al*, 1972; Muggia *et al*, 1972). These observed urological toxicities might be explained by the acidic environment in the bladder which favours the formation of the insoluble lactone form of camptothecin. Patients at the highest dose level (130 mg m^−2^ MAG CPT) were treated with sodium bicarbonate after onset of their symptoms with minor result. A potential better approach to overcome this toxic effect is to alkalise the urine by treating the patients concomitantly with sodium bicarbonate. A drawback of this approach is that MAG-CPT circulates in the body for a relatively long time and this will mean that patients should be alkalinised during that time. Patients experiencing complaints of dysuria had a significant higher AUC_0–96_ of bound camptothecin indicating that renal toxicity often encountered after one or more courses might be predicted by pharmacokinetic parameters derived in the first course. Two markers of renal function (potassium and urea) were found to be clinical indicators of variance in free camptothecin, but not in bound camptothecin. This provides preliminary evidence for altered pharmacokinetics of MAG-CPT in patients with impaired renal function.

Pharmacokinetics showed dose proportionality of bound and free camptothecin. A linear relationship was observed for the AUC_∞_ of bound (*r*=0.83, *P*<0.002) camptothecin with dose (mg m^−2^). Also free camptothecin showed a significant linear correlation (*r*=0.61, *P*<0.025) with dose (mg m^−2^) in spite of high interpatient variation in AUC_∞_ at the higher dose levels. No difference in terminal half-lives of bound and free camptothecin or clearance of bound camptothecin was found between dose levels. The administered dose was mainly excreted by the kidneys and 69±18% (mean±s.d.) of the dose was recovered in the urine within 4 days. The half-life of free camptothecin was considerably increased (11.2±9.4, mean±s.d.) compared to conventionally administered camptothecin (Rivory and Robert, 1995; Chabot, 1997). The half-lives of bound and free camptothecin were equal indicating that the kinetics of free camptothecin was dependent of the release rate from its polymer carrier. In (pre-) clinical studies with similar drug-polymer constructs a prolonged presence of (free) drug in plasma could also be demonstrated (Caiolfa *et al*, 2000). This increased half-life might be of potential benefit because camptothecin and analogues are found to be cell-cycle specific drugs and prolonged exposure could increase anti-tumour activity (Kingsbury *et al*, 1990; Houghton *et al*, 1995). As a result of their relatively long half-life both bound and free camptothecin accumulation takes place in plasma during the 3 days of administration and detectable levels of drug were observed in urine and plasma of most patients just prior the next administration. This could be an explanation for the seemingly accumulated dose related toxicity observed in some patients. Hence, toxicity might also be avoided by increased time intervals between dosing, or by administration of a single dose. The ratio of free and bound camptothecin is not significantly different between dose-levels, however there is a trend that this ratio increases with increasing dose possibly indicating a possible induction in involved esterolytic enzymes.

In summary we conclude that the pharmacokinetics of camptothecin are substantially changed by binding it to a polymeric backbone, showing controlled prolonged exposure of camptothecin. Haematological toxicity was relatively mild, but we encountered serious sustained bladder toxicity. Further investigation with MAG-CPT is warranted, but with other dosing schedules and/or with protective measures against bladder toxicity.
